# Competition between Osmotic Squeezing versus Friction-Driven Swelling of Gels

**DOI:** 10.3390/gels7030094

**Published:** 2021-07-14

**Authors:** Miyu Seii, Tomoki Harano, Masao Doi, Yoshimi Tanaka

**Affiliations:** 1 Graduate School of Environment and Information Sciences, Yokohama National University, Yokohama 2408501, Japan; nutyawuu.wk@gmail.com (M.S.); harano-tomoki-hw@ynu.jp (T.H.); 2 Department of Physics, Beihang University, Beijing 100191, China; masao.doi@buaa.edu.cn

**Keywords:** cooperative diffusion, gel dynamics, osmotic pressure

## Abstract

Some types of hydro-gels have almost the same equilibrium swelling volume in water and in ethylene glycol (EG), a highly viscous liquid completely miscible with water. Experiments showed that when a gel fully swollen with EG is immersed into a large amount of water, it temporarily swells up and then relaxes to the equilibrium volume in water. The temporary swelling is explained by the friction force exerted on the gel network from the outward EG flux In this paper, we experimentally show that the temporary swelling is suppressed by adding linear PEG (polyethylene glycol) in the outer water. Although the suppression seems to be explained by the osmotic pressure (i.e., by the same mechanism as the conventional osmotic squeezing), our theoretical analysis reveals that the effect of PEG is much stronger than that expected from the equilibrium osmotic pressure, implying that the PEG chains are condensed on the gel surface.

## 1. Introduction

In the 1970s, the concept of cooperative diffusion of gels was established [1]. According to the concept, the time change of the gel volume (swelling/deswelling) is governed by a diffusion equation, but the diffusion constant is much (roughly two digits) smaller than that of the self-diffusion of the solvent (water for hydro-gels), although the volume change is caused by solvent transport. The smaller diffusion constant is called “cooperative diffusion” constant [2]. In a sense, the cooperative diffusion reflects the collective motions of partial chains in the gel network. Distinction of the cooperative diffusion from the molecular diffusion of the solvent is often emphasized in gel science.

Recent experimental and theoretical investigations, however, have revealed that for gels swollen in binary solvents, the molecular diffusion of the solvents couples strongly with the cooperative diffusion (i.e., the volume change) of the gel network even when the equilibrium swelling volume hardly depends on the mixing ratio of the binary solvent [3,4]. When an acrylamide gel saturated with ethylene glycol (EG), say “EG-swollen gel”, is immersed into a large amount of water, its volume once increases and then decreases toward the equilibrium value even though the gel has almost the same equilibrium swelling volume in EG and in water; and the time scale of the initial temporary swelling is much faster than that expected from the cooperative diffusion constant and the gel size.

The point to understand the above-mentioned non-monotonous volume change is the asymmetry in the friction coefficients of the solvents to the polymer [4]. After the immersing of an EG-swollen gel into water, the mutual diffusion (i.e., the mixing) of inner EG and outer water occurs. The fluxes of EG and water are directed outwardly and inwardly, respectively. They exert opposite drag forces on the gel network. The drag force by EG, which has a higher viscosity, should be larger and hence the displacement of the gel network is directed into the same direction as the EG flux, i.e., outwardly. When the mutual diffusion is almost completed, the gel network starts to return to its original volume because the equilibrium swelling volume is almost the same for EG and water. The above explanation is an intuitive interpretation for the theoretical consequence of our previous investigation; theoretical details are given in the original paper [4] where an important role of the hydrostatic pressure p is also emphasized.

**Figure 1:**
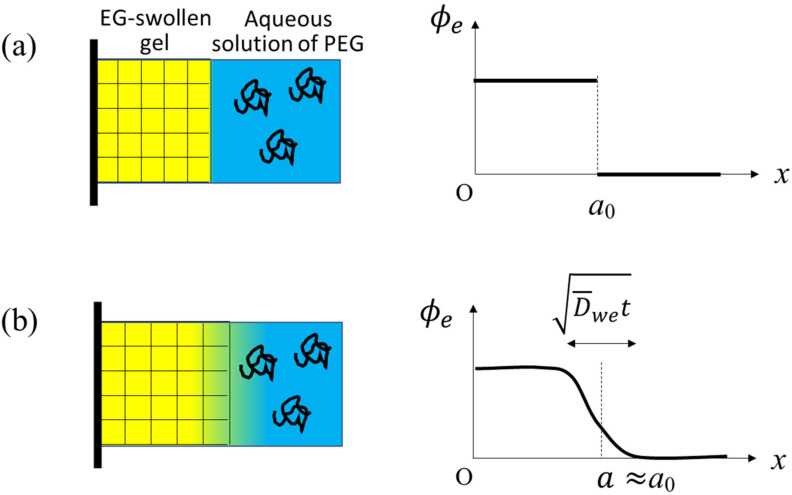
(**a**) A schematic representation of the system at the initial state of t=0 (**left**) and the initial profile of the volume fraction $\phi_{e}$ of EG, ethylene glycol (**right**). In the left illustration, the yellow and blue regions represent EG and water, respectively, and the random coils represent PEG chains. (**b**) A schematic representation of the gel (**left**) and a slightly smoothed profile of $\phi_{e}$ (**right**) at a time t during the early stage of the solvent exchange when the mixing of EG and water occurs locally and vigorously around the gel surface. ${\bar{D}}_{we}$ is a typical value of $D_{we}\left(\phi_{e}\right)$ around the gel surface.

In this paper, we investigate what happens when an EG-swollen gel is immersed into a large amount of aqueous solution of linear polyethylene glycol (PEG) chains, as illustrated in Figure 1a. The difference from the conventional osmotic squeezing experiments [5] is that the initial gel contains EG, which diffuses out when exposed to the outer aqueous solution with exerting the outward friction force on the gel network. In the conventional osmotic squeezing, the gel volume monotonously decreases with time and the dynamics of the volume reduction is governed by the cooperative diffusion. The so-called osmotic pressure II determines the extent of the volume reduction at the final equilibrium. In our case of the EG-swollen initial gel, the final equilibrium volume is identical to that in the conventional osmotic squeezing, because EG in the initial gel is substituted by water sooner or later. The transient state, however, should be different because of the competition between the outward friction force and osmosis. The question we want to address by this experimental setting is whether the quasi-equilibrium osmotic pressure concept applies to this dynamical phenomenon or not. A comparison between the experimental results and a theoretical consideration reveals that the osmotic pressure estimated from the outer PEG concentration is unsuitable for quantifying the effect of the PEG. In the early stage of the experiment, PEG chains have a stronger impact on the volume change mechanism than that expected from the equilibrium osmotic pressure.

## 2. Results and Discussion

Cylindrical (1 mm in diameter and 30 mm in length) and disk-shaped (80 mm in diameter and 20 mm in thickness) acrylamide gels were used for the solvent exchange experiment and for indentation test, respectively. The synthesis procedure of the gel (including the composition of the pre-gel solution) was identical to that in previous investigations [4,6]; see Section 4 for experimental details. The indentation test was done in order to measure the bulk mechanical property of the specimen gel and to check the consistency with the osmotic property; the used indenter is a stainless steel ball with a radius of R=6mm.

**Figure 2:**
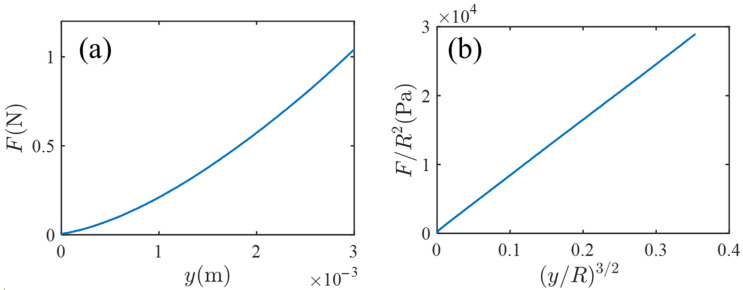
(**a**) A plot of the measured indentation force F versus indentation length y in the indentation experiment where a rigid spherical indenter is pushed against a thick gel disk. (**b**) The reduced plot based on the prediction of the Hertz contact theory.

Figure 2 shows the result of the indentation test. Figure 2a is a plot of the measured indentation force F versus indentation length y. Figure 2b is the reduced plot of ($F/R^{2})$ versus ${\left(\frac{y}{R}\right)}^{3/2}$; the choice of the quantities of the vertical and horizontal axes is based on the prediction of the Hertz contact theory [7], $F=\frac{4E}{3\left(1-\nu^{2}\right)}R^{1/2}y^{3/2}$, where E and ν are the Young modulus and the Poisson ratio, respectively. In Figure 2b, the linearity is fine, and from the slope of Figure 2b (and setting ν=1/2), the Young modules E is estimated by $E\approx 4.49\times 10^{4}$ Pa.

**Figure 3:**
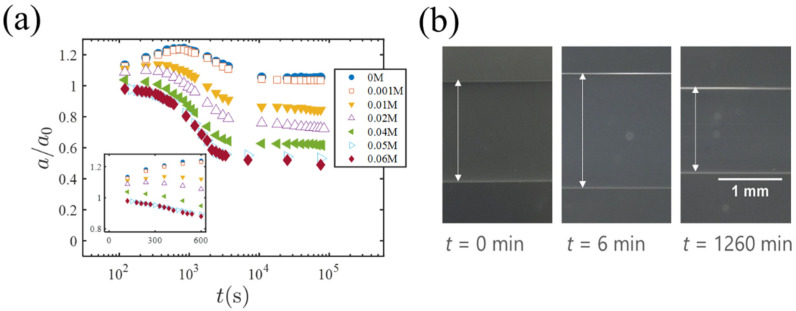
(**a**) The time change of the gel radius a(t) (normalized by the initial radius $a_{0}=a\left(0\right)$) during the solvent exchange. The different plot symbols represent different PEG concentrations in the outer solution. The insert is a linear plot showing the short-time behavior. (**b**) Photos of a gel specimen ($C_{\mathrm{PEG}}=0.01$ M) at different times.

Blow, we present results of the solvent exchange experiment in which EG-swollen cylindrical gels are immersed into PEG solutions; hereafter, the concentration of PEG is denoted by $C_{\mathrm{PEG}}$. Figure 3a is a plot of the gel radius a (normalized by the initial radius $a_{0}$) versus t (t=0 is the time when the EG-swollen gel is exposed to the PEG solution). The insert shows the short-time behavior. Figure 3b shows photos of a gel specimen ($C_{\mathrm{PEG}}=$ 0.01 M) at different times. For low PEG concentrations ($C_{\mathrm{PEG}}$ = 0 and 0.001 M), the radius a(t) shows a clear peak and then decreases to a final value almost the same as (but slightly larger than) $a_{0}$. With increasing $C_{\mathrm{PEG}}$, the peak becomes lower and almost flat at a higher concentration of $C_{\mathrm{PEG}}=0.04$ M. For $C_{\mathrm{PEG}}=0.05$ and 0.06 M, it is almost certain that the peak of the gel volume does not occurs (see the insert of Figure 3a).

**Figure 4:**
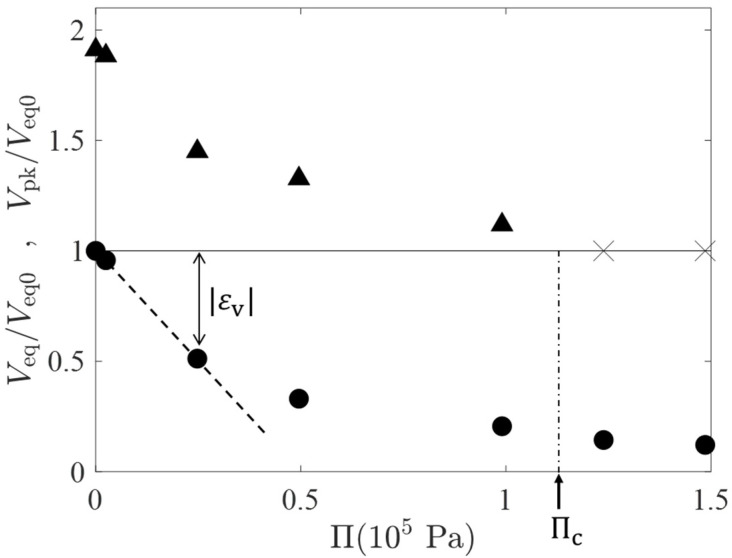
A plot of the osmotic pressure Π estimated by the van’t Hoff equation ($\Pi =RTC_{\mathrm{PEG}}$) versus the peak volume $V_{pk}$ (the filled triangles) during the temporal swelling and the equilibrium volume $V_{eq}$ (filled circles) normalized by $V_{eq0}$, the equilibrium gel volume for Π=0. The cross marks in the horizontal line of a height of unity represent that the volume peak was not observed at the Π values.

Figure 4 shows a plot of the osmotic pressure Π estimated by the van’t Hoff equation ($\Pi =RTC_{\mathrm{PEG}}$; the unit of $C_{\mathrm{PEG}}$ is converted to mol/m^3^) versus the peak volume $V_{pk}$ (the filled triangles) during the temporal swelling and the equilibrium volume $V_{eq}$ (filled circles) estimated by $a\left(t=10^{5}\;s\right)$. In Figure 4, those characteristic volumes are normalized by $V_{eq0}$, the equilibrium volume for Π=0 (i.e., $C_{\mathrm{PEG}}=0$). The cross marks in the horizontal line of a height of unity represent that the volume peak was not observed at the Π values. From the behavior of the peak volume, we have an estimation for the critical osmotic pressure $\Pi_{c}$ at which the volume peak just disappears, $\Pi_{c}\approx 1.1\times 10^{5}$ Pa. $\varepsilon_{v}≔V_{eq}/V_{eq0}-1$ represents the volumetric strain at the final equilibrium (see the double-headed arrow in Figure 4) and the initial slope of $\left|\varepsilon_{v}\right|$ versus Π relation (the dashed line) gives an estimation for the osmotic bulk modulus $K_{os}$ of $K_{os}=5.1\times 10^{4}$ Pa; $K_{os}$ is of the same order of E and approximately a half of $\Pi_{c}$. The $\varepsilon_{v}$-Π relation deviates from the linear one when $\left|\varepsilon_{v}\right|$ exceeds 0.5. This is probably due to the strong repulsion between partial chains of the highly compressed gels.

In what follows, we discuss the experimental results based on the theoretical model developed in our previous investigation [4]. We introduce a simple modification to the theoretical setting, that is, the PEG in the outer solution exerts a constant (i.e., independent of time and position) osmotic pressure Π on the gel surface.

We suppose a 1-dimentional gel (i.e., gel slab) upon the solvent exchange from EG to water (see the illustrations in Figure 1). The left surface (x=0) of the gel slab is fixed to a rigid wall and the other surface ($x=a_{0}$) is exposed to the outer solution. Actual specimens used in the experiment were cylindrical, and at the central axis of the cylinder, the radial components of the displacement of the gel network and solvent fluxes are zero by symmetry. The rigid wall in the present theoretical setting corresponds to the central axis.

The Onsager principle gives a set of time-evolutional equations of the gel dynamics in the binary solvent [4] with the aid of the conservation laws:

(1) $$\begin{array}{c} \frac{\partial \phi_{i}}{\partial t}=-\frac{\partial \left(\phi_{i}v_{i}\right)}{\partial x}\left(i=w,e,p\right) \end{array}$$

where $\phi_{i}\left(x,t\right)$ and $v_{i}\left(x,t\right)$ are the volume fraction and velocity of the *i*-th component, respectively (subscripts of “w”, “e” and “p” represent water, EG and polymer (of the gel network), respectively). Note that $v_{p}=\frac{\partial u\left(x,t\right)}{\partial t}=\dot{u}\left(x,t\right)$, where u(x,t) is the displacement of the gel network from the initial and equilibrium position (hereafter, we often use the dot notation for time derivatives). The Onsager principle in the present case is symbolically expressed as ${\left(\frac{\delta R}{\delta v_{i}}\right)}_{\phi_{i}}=0$, where R is the Rayleighian defined as the sum of a half of the dissipation rate Φ of the entire system and the time derivative of the system energy $\frac{dA}{dt}=\dot{A}$. The above symbolic expression represents the stationary condition for R with respective to a small change of $v_{i}$ at fixed $\phi_{i}$. We employ the following forms of Φ and A

(2) $$\begin{array}{c} \Phi =\frac{1}{2}\int dx\underset{i,j=w,e,p}{\sum }\zeta_{ij}\phi_{i}\phi_{j}{\left(v_{i}-v_{j}\right)}^{2} \end{array}$$

(3) $$\begin{array}{c} A=\int dx\left[f_{mix}\left(\phi_{w},\phi_{e}\right)+\frac{k}{2}{\left(\frac{\partial u}{\partial x}\right)}^{2}\right]+\Pi u\left(a_{0},t\right) \end{array}$$

where $\zeta_{ij}$ is the friction coefficient per volume (and per unit volume fractions) between the i-j combination; $f_{mix}=\frac{k_{B}T}{{\bar{V}}_{s}}\left(\phi_{w}\ln\phi_{w}+\phi_{e}\ln\phi_{e}\right)$ is the mixing free energy density (where ${\bar{V}}_{s}$ is the mean molecular volume of the solvents; we consider the entropic term only as in [4], because the equilibrium swelling volume of the gel hardly depends on $\phi_{e}$, the solvent composition) and k is the elastic modulus of the gel network. The second term of Equation (3), corresponding to the “*pV* term” in the enthalpy of gases, comes from the assumption that the effect of PEG in the outer solution is exerting a constant osmotic pressure Π on the surface of the gel network at $x=a_{0}$ (and the left surface is fixed to the rigid surface, u(0,t)=0). Because of the incompressibility of the system, $\phi_{w}+\phi_{e}+\phi_{p}=1$, the quantity to be minimized is $\tilde{R}=\Phi +\dot{A}+\int dxp\left(x,t\right)\left({\dot{\phi }}_{w}+{\dot{\phi }}_{e}+{\dot{\phi }}_{p}\right)$, where the Lagrange multiplier p(x,t) has the physical meaning of pressure. The integral terms contained in $\tilde{R}$ are completely identical to those in [4]. Thus, a set of time-evolutional equations completely identical to those in [4] are obtained by the parallel procedure of calculation; that is, (i) replacing ${\dot{\phi }}_{i}$ with $v_{i}$ by use of the conservation law of Equation (1) and of integration by parts; (ii) calculating the variation of $\delta \tilde{R}$ with respect to $\delta v_{i}$ to obtain the relations between the velocities $v_{i}$ and the thermodynamical potentials $\mu_{i}=\frac{\partial f_{mix}}{\partial \phi_{i}}+p$; and (iii) re-employing Equation (1) to obtain the closed set of equations. When the gel network is dilute but its deformation remains small, the process of the solvent mixing is hardly disturbed by the gel network. In this case, a perturbative consideration allows us to simplify a complicated set of coupled partial differential equation of the gel dynamics into a pair of diffusion equations describing the mixing of the solvents

(4) $$\begin{array}{c} \frac{\partial \phi_{e}}{\partial t}=-\frac{\partial }{\partial x}j_{e}=\frac{\partial }{\partial x}[D_{we}\left(\phi_{e}\right)\frac{\partial \phi_{e}}{\partial x}] \end{array}$$

and the force balance condition for a unit volume of the gel network

(5) $$\begin{array}{c} \phi_{p0}\zeta_{s}\left(\phi_{e}\right)\frac{\partial u}{\partial t}=k\frac{\partial^{2}u}{\partial x^{2}}+\phi_{p0}\left(\zeta_{ep}-\zeta_{wp}\right)\left(-D_{we}\left(\phi_{e}\right)\frac{\partial \phi_{e}}{\partial x}\right) \end{array}$$

(6) $$\begin{array}{c} \zeta_{s}\left(\phi_{e}\right)=\zeta_{ep}\phi_{e}+\zeta_{wp}\phi_{w}=\zeta_{ep}\phi_{e}+\zeta_{wp}\left(1-\phi_{e}\right) \end{array}$$

where $D_{ew}\left(\phi_{e}\right)$ is the mutual diffusion coefficient of the solvents, $j_{e}=-D_{we}\left(\phi_{e}\right)\frac{\partial \phi_{e}}{\partial x}$ is the volume flux of EG, and $\phi_{p0}$ is the polymer volume fraction in the initial and the final equilibrium state, and $\zeta_{s}\left(\phi_{e}\right)=\zeta_{ep}\phi_{e}+\zeta_{wp}\phi_{w}=\zeta_{ep}\phi_{e}+\zeta_{wp}\left(1-\phi_{e}\right)$ is the friction coefficient of the ‘means’ solvent. The term proportional to $\left(\zeta_{ep}-\zeta_{wp}\right)$ in Equation (5) represents the friction force by the solvent fluxes.

On the other hand, the osmotic pressure term $\Pi u\left(a_{0},t\right)$ in Equation (3) plays an important role in the boundary condition on the right gel surface. That is, by using integration by parts, $\dot{A}=\int dx\left[\underset{i,j=w,e}{\sum }\frac{\partial f_{mix}}{\partial \phi_{i}}{\dot{\phi }}_{i}-\left(k\frac{\partial^{2}u}{\partial x^{2}}\right)\dot{u}\right]+\left(k\frac{\partial u}{\partial x}\left(a_{0},t\right)+\Pi \right)\dot{u}\left(a_{0},t\right)$ and the variation of the second term leads to the following boundary condition on the gel surface,

(7) $$\begin{array}{c} k\frac{\partial u}{\partial x}\left(a_{0},t\right)+\Pi =0 \end{array}$$

Note that in the present simplified (perturbative) treatment, any boundary (or connection) condition of $\phi_{e}$ is not imposed on the gel surface, because we assume that the mixing of the solvents occurs as if the gel network does not exist.

Based on Equations (4)–(7), we consider the critical osmotic pressure $\Pi_{c}$ at which the peak of the gel volume just disappears. At $\Pi =\Pi_{c}$, the gel surface is stationary in the early stage of the solvent exchange (or solvent mixing); we may consider that the inside of the gel is also stationary, $\dot{u}\left(x,t\right)\approx 0$ for $0<x<a_{0}$. With this simplification, the force balance condition of Equation (5) is reduced to $-k\frac{\partial^{2}u}{\partial x^{2}}\approx \phi_{p0}\left(\zeta_{ep}-\zeta_{wp}\right)\left(-D_{we}\left(\phi_{e}\right)\frac{\partial \phi_{e}}{\partial x}\right)$. Integrating both sides from x=0 to $a_{0}$ and using Equation (7), we obtain ${\left[-k\frac{\partial u}{\partial x}\right]}_{0}^{a_{0}}\approx \Pi_{c}\approx \phi_{p0}\left(\zeta_{ep}-\zeta_{wp}\right)\int_{0}^{a_{0}}dxD_{we}\left(\phi_{e}\right)\left(-\frac{\partial \phi_{e}}{\partial x}\right)>\phi_{p0}\left(\zeta_{ep}-\zeta_{wp}\right)D_{we}\left(1\right){\left[-\phi_{e}\left(x\right)\right]}_{0}^{a_{0}}$, where $D_{we}\left(1\right)=D_{we}\left(\phi_{e}\to 1\right)$ is the mutual diffusion coefficient in the EG-rich limit. In the first approximative equality, we use $\frac{\partial u}{\partial x}\left(0,t\right)\approx 0$ in the early stage of the solvent mixing (see Figure 1b) and the rightmost inequality comes from the fact that $D_{ew}\left(\phi_{e}\right)$ decreases with $\phi_{e}$. Because ${\left[-\phi_{e}\left(x\right)\right]}_{0}^{a_{0}}$ is 1/2 or so, we have an order estimation for the critical osmotic pressure:

(8) $$\begin{array}{c} \Pi_{c}∼\phi_{p0}\left(\zeta_{ep}-\zeta_{wp}\right)D_{we}\left(1\right) \end{array}$$

The right-hand side of Equation (8) can be estimated by relating $\zeta_{ep}$ and $\zeta_{wp}$ to the cooperative diffusion constants in pure EG and pure water. Setting $\phi_{e}=0$ and 1 in Equations (5) and (6), we have $\frac{\partial u}{\partial t}=D_{co,i}\frac{\partial^{2}u}{\partial x^{2}}\left(i=w,e\right)$, where $D_{co,w}=\frac{k}{\zeta_{ew}\phi_{p0}}$ and $D_{co,e}=\frac{k}{\zeta_{ep}\phi_{p0}}$ are the cooperative diffusion constants of the gel in water and in EG, respectively, and $\frac{\zeta_{ep}}{\zeta_{ew}}=\frac{D_{co,w}}{D_{co,e}}$. Because the equilibrium volume of the gel (or the mesh size of the gel network) is almost the same for water and for EG, $\frac{D_{co,w}}{D_{co,e}}$ is governed by the ratio of viscosities of water ($\eta_{w}$) and EG ($\eta_{e}$). Hence, $\frac{\zeta_{ep}}{\zeta_{ew}}=\frac{D_{co,w}}{D_{co,e}}=\frac{\eta_{e}}{\eta_{w}}$. At room temperature, the viscosity ratio is $\frac{\eta_{e}}{\eta_{w}}\approx 16$. Combining the above observation, $\zeta_{ep}-\zeta_{wp}\approx \zeta_{ep}$ and $\zeta_{ep}=\frac{k}{\phi_{p0}D_{co,e}}=\frac{k}{\phi_{p0}D_{co,w}}\frac{\eta_{e}}{\eta_{w}}$. Thus, Equation (8) becomes $\Pi_{c}∼k\frac{D_{we}\left(1\right)}{D_{co,w}}\frac{\eta_{e}}{\eta_{w}}$. According to literature, $D_{co,w}\approx 2.1\times 10^{-11}$ m/s^2^ [6] and $D_{we}\left(1\right)\approx 3.0\times 10^{-10}$ m^2^/s [8]. We may consider $k∼K_{os}\approx 0.5\times 10^{5}$ Pa. Thus, we have $\Pi_{c}∼10^{7}$ Pa. This is, however, much (2 digits) larger than the experimental value of $\Pi_{c}$ estimated by the van’t Hoff equation. The above theoretical consideration says that the “effective osmotic pressure” in the early stage of the solvent exchange is much higher than the equilibrium one.

Why can the PEG chains in the outer solution suppress the friction-driven volume expansion so effectively? One may suppose that the outward EG flux exerts friction forces on the PEG chains, as well as the gel network, to drive them away from the gel surface (and the osmotic pressure by PEG is screened). This is opposite to what actually occurs. A possible answer for the above question is the affinity (i.e., the enthalpic interactions) among the components, which has not been taken into account in our coupled diffusion model. Because of the similarity in the chemical structure, PEG chains may have a stronger affinity to EG than that to water and be attracted by the EG secreted on the gel surface to form a condensation layer. If the condensation layer is actually formed, it could strongly suppress the friction-driven swelling by localized enhancement of the osmotic pressure around the gel surface and/or by a sort of masking effect that weakens the outward EG flux. To judge the validity of this conjecture, further experimental, theoretical and numerical investigations are needed.

## 3. Conclusions

The relation between diffusion and osmosis (occurrence of stress and/or convectional transport driven by mixing entropy) is a historically important subject discussed by Einstein in his theory of the Brownian motion [9] and also current topics [10] linked to several research fields such as physiology [11], environmental engineering [12] and non-equilibrium and soft matter physics [13]. This study clearly shows that in the strongly non-equilibrium state of solvent exchange of gels, the thermodynamical osmotic pressure is not a useful concept. Gels always raise interesting questions in ‘Osmology’.

## 4. Materials and Methods

In the solvent exchange experiment, cylindrical gels (1 mm in diameter and 30 mm in length) were used. The synthesis procedure (including the composition of pre-gel solution) was identical to that in the previous investigations [4,6]. The as-prepared gels were immersed into a large amount of distilled water for three days in order to remove reaction residuals, and then moved into a bath of EG for two weeks in order to exchange the inner solvent. Next, an EG-swollen gel was moved into a specimen cell filled with aqueous solution of PEG (Mw = 5000; FUJIFILM Wako Pure Chemical Co., Osaka, Japan). The PEG concentration $C_{\mathrm{PEG}}$ changes from 0 (pure water) to 0.06 M. The time change of the gel radius a(t) was observed with a digital microscope (VHX600; Keyence Co., Osaka, Japan) and measured on the recorded images (*t* = 0 is the time when the gel is moved into the PEG solution). During the very early stage of the solvent exchange, t≤100 s, the microscope images were indistinct and a(t) was not able to be measured. This is probably because the EG secreted from the initial gel forms a thin diffusion layer where the EG-water composition (i.e., the refraction index) steeply changes with position (i.e., the distance from the gel surface) and the diffusion layer prohibits the formation of sharp images of the gels.

In the indentation test, disk-shaped gels with 20 mm in thickness and 80 mm in diameter were used. The test was carried-out on the as-prepared gels with an indentation resistance tester, TA.XT plus (Stable Micro System Ltd., Surrey, UK); the used indenter is a stainless steel ball with a radius of R=6 mm. The indentation rate was 0.1 and 1 mm/s, but there was no notable difference in the measured force curves (and estimated the Young modulus) for these indentation rates.

## References

[1] Tanaka T., Fillmore D.J. (1979). Kinetics of swelling of gels. J. Chem. Phys..

[2] de Gennes P.-G. (1979). Scaling Concepts in Polymer Physics.

[3] Toyotama A., Sawada T., Yamanaka J., Kitamura K. (2006). Optical Detection of nonequilibrium swelling behavior of a polymer gel upon solvent substitution. Langmuir.

[4] Tanaka Y., Seii M., Sui J., Doi M. (2020). Gel dynamics in the mixture of low and high viscosity solvents: Re-entrant volume change induced by dynamical asymmetry. J. Chem. Phys..

[5] Bastide J., Candau S., Leibler L. (1981). Osmotic deswelling of gels by polymer solutions. Macromolecules.

[6] Tokita M., Tanaka T. (1991). Friction coefficient of polymer networks of gels. J. Chem. Phys..

[7] Johnson K.L. (2008). Contact Mechanics.

[8] Ternström G., Sjöstrand A., Aly G., Jernqvist A. (1996). Mutual diffusion coefficients of water+ ethylene glycol and water+ glycerol mixtures. J. Chem. Eng. Data..

[9] Einstein A. (1956). Investigations on the Theory of the Brownian Movement.

[10] Marbach S., Bocquet L. (2019). Osmosis, from molecular insights to large-scale applications. Chem. Soc. Rev..

[11] Mitchison T.J., Charras G.T.L. (2008). Mahadevan, Implications of a poroelastic cytoplasm for the dynamics of animal cell shape. Semin. Cell Dev. Biol..

[12] Hoover L.A., Phillip W.A., Tiraferri A., Yip N.Y., Elimelech M. (2011). Forward with osmosis: Emerging applications for greater sustainability. Environ. Sci. Technol..

[13] Doi M. (2013). Soft Matter Physics.

